# CD206 Expression in Induced Microglia-Like Cells From Peripheral Blood as a Surrogate Biomarker for the Specific Immune Microenvironment of Neurosurgical Diseases Including Glioma

**DOI:** 10.3389/fimmu.2021.670131

**Published:** 2021-06-29

**Authors:** Shunya Tanaka, Masahiro Ohgidani, Nobuhiro Hata, Shogo Inamine, Noriaki Sagata, Noritoshi Shirouzu, Nobutaka Mukae, Satoshi O. Suzuki, Hideomi Hamasaki, Ryusuke Hatae, Yuhei Sangatsuda, Yutaka Fujioka, Kosuke Takigawa, Yusuke Funakoshi, Toru Iwaki, Masako Hosoi, Koji Iihara, Masahiro Mizoguchi, Takahiro A. Kato

**Affiliations:** ^1^ Department of Neurosurgery, Graduate School of Medical Sciences, Kyushu University, Fukuoka, Japan; ^2^ Department of Neuropsychiatry, Graduate School of Medical Sciences, Kyushu University, Fukuoka, Japan; ^3^ Department of Neuropathology, Graduate School of Medical Sciences, Kyushu University, Fukuoka, Japan; ^4^ Department of Psychosomatic Medicine, Kyushu University Hospital, Fukuoka, Japan

**Keywords:** microglia, glioma, CD206, surrogate biomarker, induced microglia-like cells

## Abstract

Targeting the unique glioma immune microenvironment is a promising approach in developing breakthrough immunotherapy treatments. However, recent advances in immunotherapy, including the development of immune checkpoint inhibitors, have not improved the outcomes of patients with glioma. A way of monitoring biological activity of immune cells in neural tissues affected by glioma should be developed to address this lack of sensitivity to immunotherapy. Thus, in this study, we sought to examine the feasibility of non-invasive monitoring of glioma-associated microglia/macrophages (GAM) by utilizing our previously developed induced microglia-like (iMG) cells. Primary microglia (pMG) were isolated from surgically obtained brain tissues of 22 patients with neurological diseases. iMG cells were produced from monocytes extracted from the patients’ peripheral blood. Quantitative reverse transcription-polymerase chain reaction (qRT-PCR) revealed a significant correlation of the expression levels of representative markers for M1 and M2 microglia phenotypes between pMG and the corresponding iMG cells in each patient (Spearman’s correlation coefficient = 0.5225, *P <*0.0001). Synchronous upregulation of CD206 expression levels was observed in most patients with glioma (6/9, 66.7%) and almost all patients with glioblastoma (4/5, 80%). Therefore, iMG cells can be used as a minimally invasive tool for monitoring the disease-related immunological state of GAM in various brain diseases, including glioma. CD206 upregulation detected in iMG cells can be used as a surrogate biomarker of glioma.

## Introduction

Gliomas are tumors of the central nervous system (CNS) derived from neural tissues. Among them, glioblastoma (GBM) has a highly aggressive phenotype and accounts for most gliomas. Despite advances in surgical resection, chemotherapy, and radiation therapy, GBM prognosis remains poor, and <5% of patients survive beyond five years post-diagnosis (1).

Among the recent attempts to develop multimodal treatment strategies to modify the extremely poor survival of GBM patients, immune checkpoint inhibitors were expected to bring about a paradigm shift, similar to that achieved in the treatment of other malignancies, such as melanoma (2, 3). However, clinical trials have failed to observe significant therapeutic benefits of immune checkpoint inhibitors in patients with GBM (4, 5). Such unfavorable results may be partly due to the peculiar state of the immune system in glioma tissues and the CNS in general.

Microglia are the brain’s immune system cells responsible for maintaining brain homeostasis (6). Microglia exhibit a spectrum of phenotypes. The classically activated microglia/macrophages stimulate anti-tumor immune responses through the secretion of pro-inflammatory cytokines, such as tumor necrosis factor-alpha (TNF-α), interleukin (IL)-1β, and inducible nitric oxide synthase, defined as M1 markers. However, the alternatively activated microglia/macrophages promote tumor survival by producing anti-inflammatory cytokines such as IL-4, transforming growth factor-beta (TGF-β), and IL-10, defined as M2 markers (7–10). Microglia and peripheral macrophages recruited by glioma cells, defined as glioma-associated microglia/macrophages (GAM), were shown to contribute to tumor growth and invasion (11).

GBM is a complex solid tumor containing neoplastic and non-neoplastic cells, and the majority of the non-neoplastic cells are GAM, which account for 30%-50% of the cells in GBM (12, 13). GAM have been reported to play various roles in the malignancy features of GBM, including proliferation, growth, invasion, and immunosuppression (14–17). GAM are recruited to GBM microenvironment, where they release a wide array of chemokines and cytokines in response to the factors secreted by the tumor cells (14). Recently, small extracellular vesicles secreted by GAM have been reported to promote the progression of glioma (18). Within the tumor microenvironment, GAM are forced to transform to M2 phenotypes by GBM cells that secrete factors such as IL-10, IL-4, IL-6, macrophage colony-stimulating factor, macrophage inhibitory factor, TGFβ, and prostaglandin E_2_, which subsequently supports tumor growth and invasion (19). Several studies of tumor tissue samples have documented a correlation between GAM characteristics and pathological grade/prognosis of gliomas (20, 21).

We have previously developed a technique to generate induced microglia-like (iMG) cells from peripheral blood (22). We reported that iMG cells expressed the essential characteristics of human microglia, such as surface markers and drug responses, phagocytosis, and cytokine production (22). We applied this technique for translational research focusing on neurological, psychiatric, and pain-related disorders (23, 24). Other research groups and ours have revealed that iMG cells express microglia-specific surface markers (CX3CR1, P2RY12, TMEM119, and others) and possess phagocytic activity (22, 25, 26). Recently, iMG cells have been reported to be distinct from monocytes and macrophages but clustered closer with human brain microglia [Ohgidani 2020 under review] (25). These results suggest that iMG cells are a promising research tool for less-invasive monitoring of the immunological state of microglia in the CNS.

CD206 is a 175 kDa transmembrane protein encoded by the mannose receptor C-type 1 gene (*MRC1*). It is mostly expressed in macrophages, dendritic cells, and endothelial cells, where it functions as a receptor for mannosylated ligands, such as microbial antigens (27). In neural tissues, expression of CD206 is observed in microglia (28, 29) and astrocytes (30, 31). CD206 is widely recognized as a representative M2 microglial marker (29, 32). CD206 is involved in important cellular functions, especially in pinocytosis and phagocytosis (28, 31, 33). Therefore, CD206 is suggested to play a critical role in the first step of the recognition and capture of pathogens in neural tissues (31). A recent study has suggested a positive correlation between the World Health Organization pathological grades and the numbers of CD206-positive GAM in human glioma tumor tissues (21). Interestingly, we reported that CD206 expression in iMG cells was downregulated in patients with bipolar disorder during the manic state (34). Based on that study, we proposed that iMG cells could be candidate surrogate cells to monitor the immune environment that comprises other CD206-expressing cells in CNS diseases.

We hypothesized further that iMG cells from patients with glioma might reflect the immune properties of GAM and thereby serve as a novel biomarker of glioma. To clarify this hypothesis, we compared the immunological states of blood-derived iMG cells with those of brain-derived microglia. Further, we investigated the specificity of biological properties of these cells, which were isolated from glioma patients.

## Materials and Methods

### Patients

Microglia were isolated from the residual resected brain tissue after sampling for the pathological diagnosis from the surgical removal of subcortical or deeply located mass lesions (N = 15) or following epileptic surgery (N = 7) in a total of 22 patients (**Table 1**). During surgeries for the removal of nine gliomas (grade II, N = 2; grade III, N = 2; grade IV, N = 5) and six radiographic glioma-like mass lesions (meningioma, N = 1; metastatic tumor, N = 2; brain abscess, N = 1; encephalitis, N = 1; radiation necrosis, N = 1), the samples for microglia isolation were obtained from the surgical corridor during the approach to the lesion. In five of the seven patients with epilepsy, sampling for microglia isolation was performed at the resected epileptogenic lesion detected using chronic subdural electrodes placed during a prior surgery 1 or 2 weeks before. Resected brain tissues were immediately placed on ice and transferred to the laboratory for microglia isolation within 2 h of resection.

**Table 1:** Summary of the pathological characteristics of samples and period from subdural electrode placements.

Case No.	Sex	Diagnosis	since placement of subdural electrodes	inflammatory infiltrates due to subdural electrode placements	collect blood for Induction of iMG cells
1	Male	Tuberous sclerosis	none	n/a	−
2	Male	Epilepsy (Old cerebral contusion)	14 days	−	+
3	Female	Epilepsy	none	n/a	+
4	Male	Epilepsy	14 days	+	−
5	Male	Focal cortical dysplasia	14 days	+	+
6	Male	Focal cortical dysplasia	9 days	+	+
7	Male	Focal cortical dysplasia	7 days	−	−
8	Male	Secretory meningioma	none	n/a	+
9	Female	Radiation necrosis (chronic encapsulated expanding hematoma)	none	n/a	+
10	Male	Suspected encephalitis	none	n/a	+
11	Male	Brain abscess	none	n/a	+
12	Female	Metastatic brain tumor	none	n/a	+
13	Female	Metastatic brain tumor	none	n/a	+
14	Female	Diffuse astrocytoma (WHO grade II)	none	n/a	+
15	Male	Diffuse astrocytoma (WHO grade II)	none	n/a	+
16	Female	Anaplastic astrocytoma (WHO grade III)	none	n/a	+
17	Male	Anaplastic ganglioglioma (WHO grade III)	none	n/a	+
18	Female	Glioblastoma (WHO grade IV)	none	n/a	+
19	Female	Glioblastoma (WHO grade IV)	none	n/a	+
20	Female	Glioblastoma (WHO grade IV)	none	n/a	+
21	Male	Glioblastoma (WHO grade IV)	none	n/a	+
22	Female	Glioblastoma (WHO grade IV)	none	n/a	+

n/a, not applicable.

### Primary Microglia Isolation

**Figure 1:**
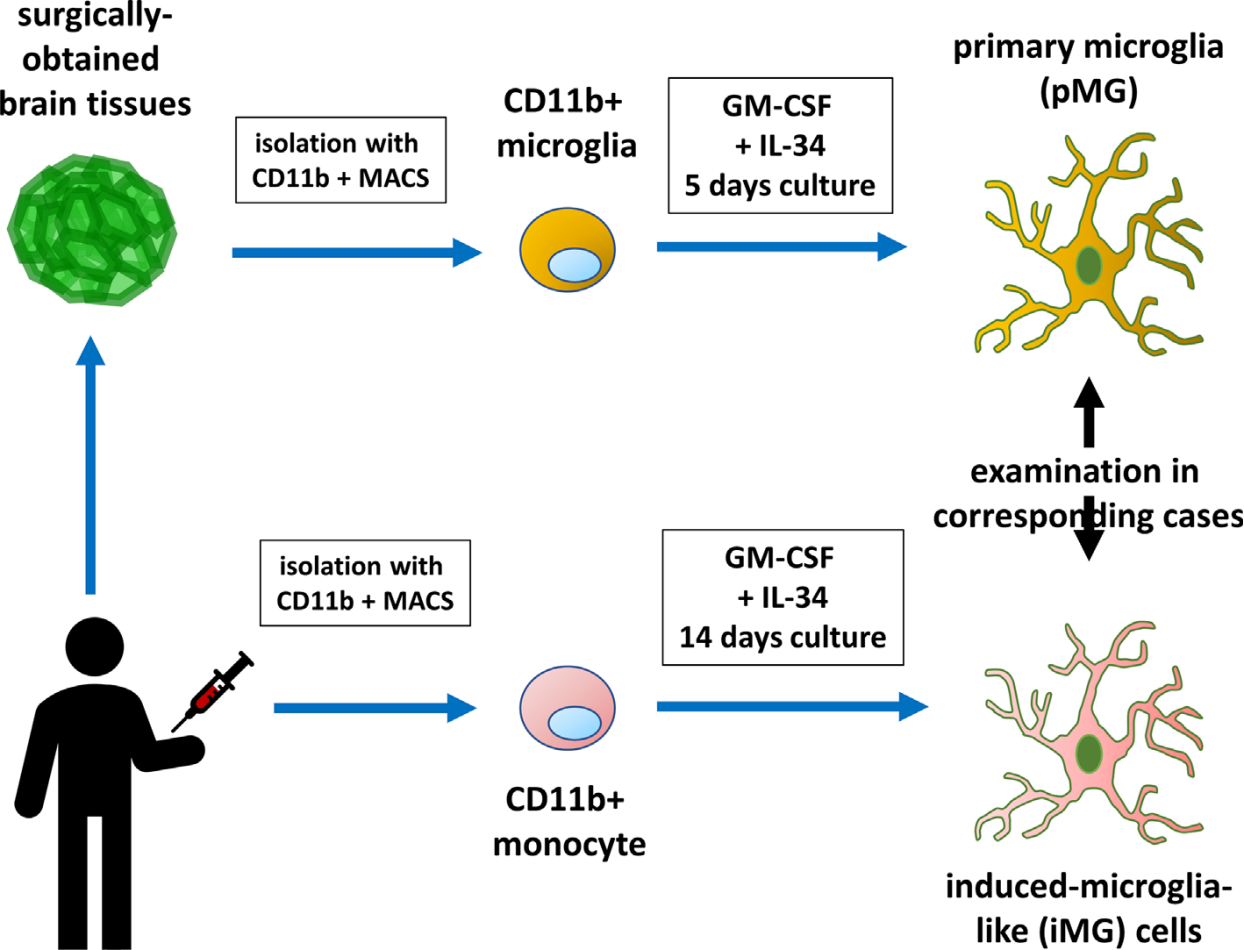
Diagram illustrating primary microglia and induced microglia-like cell collection and experiment. Monocytes isolated from the patients were induced to develop into microglia-like (iMG) cells. The biological properties of iMG cells were compared with those of brain-derived primary microglia.

Human tissue samples were dissociated using a Neural Tissue Dissociation Kit (Miltenyi Biotec, Bergisch-Gladbach, Germany), according to the manufacturer’s instructions (**Figure 1**). The cell suspension was incubated with CD11b microbeads (Miltenyi Biotec) in the MACS buffer (Miltenyi Biotec) for 15 min at 4°C. Afterward, the cells were washed, resuspended, and transferred to an LS column (Miltenyi Biotec) within a magnetic field. The positively selected (CD11b^+^) microglia were collected and resuspended in the Microglia Medium (ScienCell, Carlsbad, CA, USA). Primary microglia (pMG) were plated on culture dishes at a density of 3 × 10^5^ cells/mL and cultured overnight in standard culture conditions (37 °C, 5% CO_2_). After overnight incubation, culture supernatant and non-adherent cells were removed. Microglia were cultured in RPMI-1640 Glutamax (Invitrogen, Carlsbad, CA, USA) supplemented with 1% antibiotic/antimycotic, recombinant human granulocyte macrophage colony-stimulating factor (GM-CSF) (10 ng/mL; R&D Systems, Minneapolis, MN, USA) and recombinant human IL-34 (100 ng/mL; R&D Systems) for 5 days.

### Preparation of iMG Cells From Human Peripheral Blood

Peripheral blood was collected into a heparinized tube from patients with brain tumors, epilepsy, or other brain diseases (**Figure 1**). In three patients with epilepsy, consent to collect blood was not obtained. Peripheral blood mononuclear cells (PBMCs) were isolated using Histopaque-1077 (Sigma Chemical Co., St. Louis, MO, USA) density gradient centrifugation. PBMCs were resuspended in RPMI-1640 (Nacalai Tesque, Kyoto, Japan) supplemented with 10% heat-inactivated fetal bovine serum (Japan Bio Serum, Hiroshima, Japan) and 1% antibiotic/antimycotic (Invitrogen). PBMCs were plated on culture dishes at a density of 4 × 10^5^ cells/mL and cultured overnight in standard culture conditions (37 °C, 5% CO_2_). After overnight incubation, culture supernatant and non-adherent cells were removed. Adherent cells (monocytes) were cultured in RPMI-1640 Glutamax supplemented with 1% antibiotic/antimycotic, recombinant human GM-CSF (10 ng/mL), and recombinant human IL-34 (100 ng/mL) for 14 days to obtain iMG cells (22, 23).

### Quantitative Reverse Transcription-Polymerase Chain Reaction

To assess gene expression patterns in microglia and iMG cells, qRT-PCR was performed using a LightCycler 480 system (Roche Diagnostics, Mannheim, Germany). Microglia and iMG cells were washed. Total RNA was extracted using a High Pure RNA Isolation kit (Roche Diagnostics) according to the manufacturer’s protocol and used for cDNA synthesis using a Transcriptor First Strand cDNA Synthesis kit (Roche Diagnostics). qRT-PCR for representative markers of M1 and M2 microglia phenotypes was performed using their respective primers (**Table 2**). Normalization was performed using the reference gene glyceraldehyde-3-phosphate dehydrogenase (*GAPDH*) from the Universal ProbeLibrary (Roche Diagnostics) and the ΔΔCt method.

**Table 2:** Primer sequences used for qRT-PCR.

M1 marker	
CD80	
L	GAAGCAAGGGGCTGAAAAG
R	GGAAGTTCCCAGAAGAGGTCA
CD45	
L	AGTCAAAGTTATTGTTATGCTGACAGA
R	TGCTTTCCTTCTCCCCAGTA
HLA-DR	
L	CCCAGGGAAGACCACCTTT
R	CACCCTGCAGTCGTAAACGT
TNF-α	
L	CAGCCTCTTCTCCTTCCTGAT
R	GCCAGAGGGCTGATTAGAGA
IL-1β	
L	TACCTGTCCTGCGTGTTGAA
R	TCTTTGGGTAATTTTTGGGATCT
IL-23	
L	AGCTTCATGCCTCCCTACTG
R	CTGCTGAGTCTCCCAGTGGT
M2 marker	
CD206	
L	CACCATCGAGGAATTGGACT
R	ACAATTCGTCATTTGGCTCA
CD209	
L	AGCTGACCTGGCTGAAGG
R	GTTTCCTTGGAAGAATGTCCA
BDNF	
L	GTAACGGCGGCAGACAAA
R	GACCTTTTCAAGGACTGTGACC
CD23	
L	ACAGGAACTTGGAACAAGCAG
R	CCAGCAGCACGATCTGAGT
CCL18	
L	ATGGCCCTCTGCTCCTGT
R	AATCTGCCAGGAGGTATAGACG
IL-10	
L	GATGCCTTCAGCAGAGTGAA
R	GCAACCCAGGTAACCCTTAAA

### Immunohistochemistry

The resected brain tissues around the tumors were fixed in 10% neutral buffered formalin, embedded in paraffin, and processed for immunohistochemistry. The expression levels of the following microglia/macrophage markers were investigated in 4 μm serial paraffin sections: Iba-1 (pan-microglia/macrophage marker), CD68 (lysosomal protein; highly expressed by macrophages and activated microglia), and CD206 (mannose receptor, M2 marker). Goat antibody for Iba-1 (Cat. #ab5076, Abcam, Cambridge, MA, USA; 1:500 dilution, RRID: AB_2224402), rabbit antibody for CD206 (Cat. #ab64693, Abcam, Cambridge, MA, USA; 1:1000 dilution, RRID: AB_1523910), and mouse antibody for CD68 (Cat. #M0814, Dako, Carpinteria, CA, USA; 1:200 dilution, RRID: AB_2314148) were used as the primary antibodies. The sections were incubated with primary antibodies at 4°C overnight. Immunoreaction products were detected using the polymer immunocomplex method by an Envision system (Dako). The sections were counterstained with hematoxylin. Immunoreactivity was detected using 3,3′-diaminobenzidine (Dojindo, Kumamoto, Japan). The negative control experiments for CD 206, Iba-1, and CD68 were performed without primary antibodies (**Supplementary Figure S1**).

### Statistical Analyses

Results are expressed as the mean ± standard deviation (SD). The Spearman’s correlation coefficient was used for analyzing the correlation between parameters in pMG and iMG cells. Statistical significance was determined at α = 0.05 level. Differences were considered statistically significant when *P*-values were <0.05. Each experiment was conducted with four independent cell cultures; however, in some cases, the number of pMG or iMG cells was small and the number of samples was <4 [N = 3.68 (SD ±0.7790)].

## Results

### Gene Expression in Human GAM and iMG Cells

**Figure 2:**
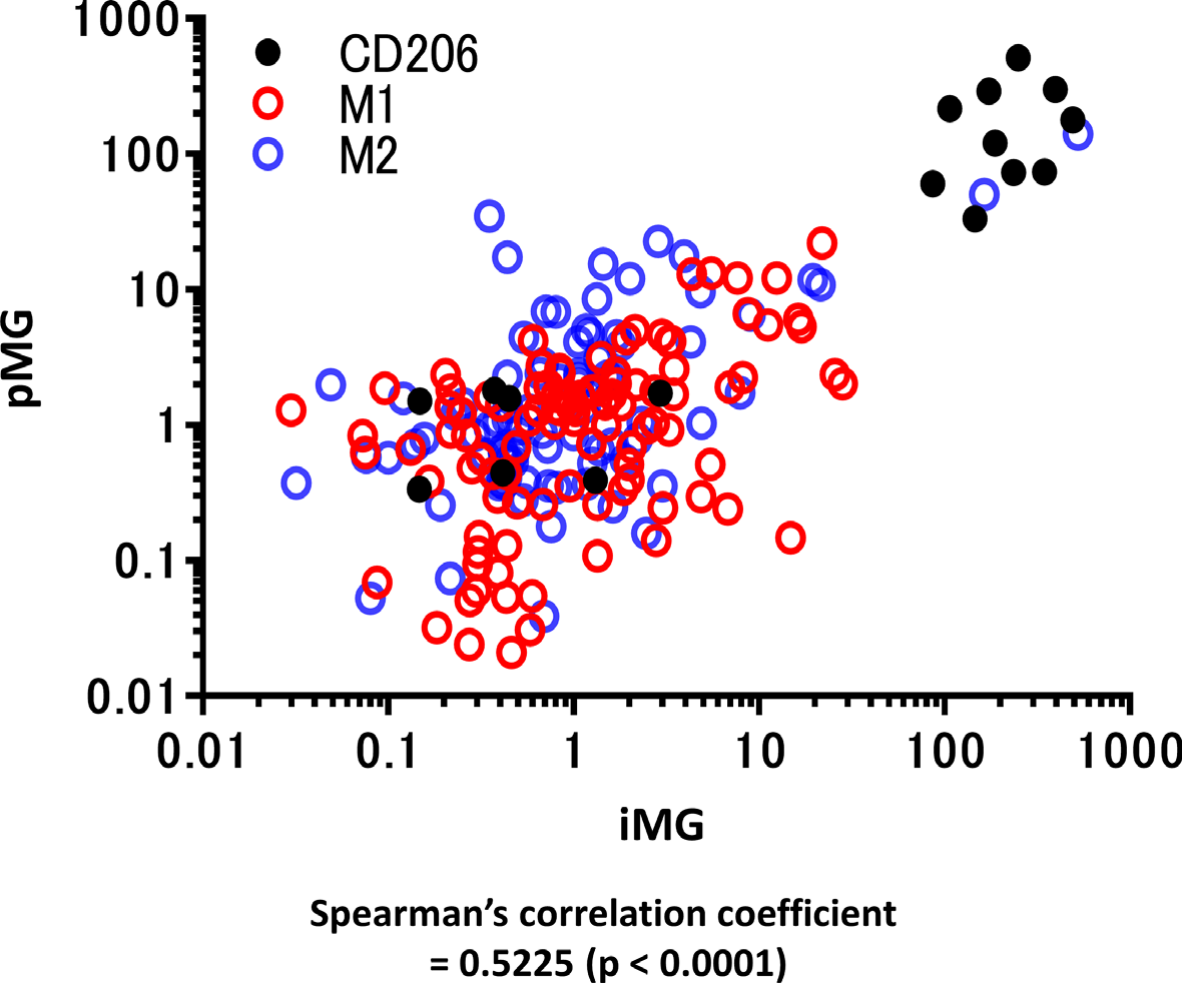
qRT-PCR for representative M1 and M2 microglial markers in primary microglia and induced microglia-like cells. mRNA levels of M1 and M2 microglia markers (12 types in total) were measured using qRT-PCR in 19 cases in which total mRNA was extracted from both induced microglia-like (iMG) cells and primary microglia (pMG). Each value was compared with the control (epilepsy cases without inflammatory infiltrates due to subdural electrode placements, N = 2) to investigate the correlation between iMG and pMG parameters. The expression levels of inflammation-related genes significantly correlated in pMG and iMG cells from the same patients (Spearman’s correlation coefficient = 0.5225, *P < *0.0001). Red circles indicate M1 markers, M2 markers are indicated by blue circles, and CD206 is indicated by black dots. qRT-PCR was performed in four independent cell cultures; however, in some cases, the number of pMG or iMG cells was small, and the number of samples was <4 [N = 3.68 (SD ±0.7790)].

Total RNA was isolated from these paired samples to investigate the expression profiles of pMG cells isolated from brain tissue and the corresponding blood-derived iMG cells. PCR was performed to determine the expression levels of inflammation-related genes known as representative markers for M1 microglia and macrophage phenotype (CD45, CD80, HLA-DR, TNF-α, IL-1β, and IL-23) and M2 phenotype (CD206, CD209, CD23, BDNF, IL-10, and CCL18) (**Supplementary Table S1**). Spearman’s correlation analysis revealed that expression levels of inflammation-related genes in the paired pMG and iMG cells derived from the same patient significantly correlated (Spearman’s correlation coefficient = 0.5225, *P* < 0.0001), indicating that iMG cells exhibited disease-related phenotypes and were regulated synchronously with the corresponding pMG (**Figure 2**).

**Figure 3:**
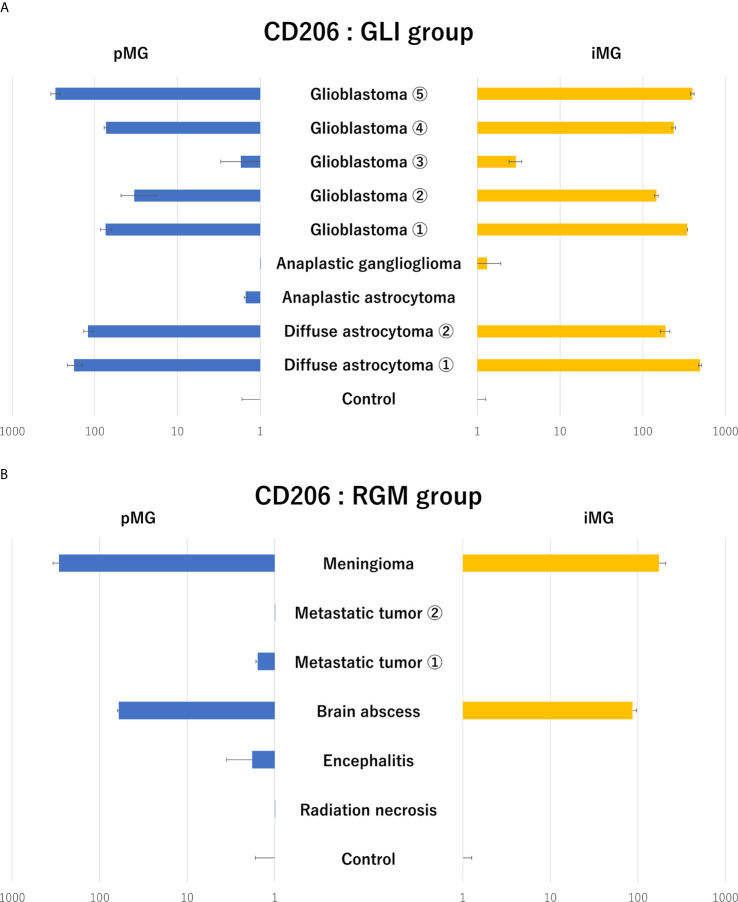
Expression levels of CD206 in primary microglia and induced microglia-like cells in patients with glioma and radiographic glioma-like mass lesions. **(A)** CD206 expression levels in primary microglia (pMG) and induced microglia-like (iMG) cells in patients with glioma (GLI group) compared to those in control samples. Synchronous upregulation in CD206 expression was detected in pMG and iMG cells in six of the nine (66%) patients, and especially in four of the five (80%) patients with GBM. **(B)** CD206 expression levels in pMG and iMG cells in patients with a radiographic glioma-like mass lesion (RGM group) compared to those in control samples. Synchronous upregulation of CD206 expression was detected in two of the six (33%) patients. We compared CD206 expression levels in the GLI and RGM groups with those from epilepsy patients as control due to the lack of samples from healthy individuals.

Next, to identify glioma-specific transcripts in GAM, the qPCR results were compared between the glioma group (GLI group; N = 9) and radiographic glioma-like mass lesion group (RGM group, N = 6; meningioma, N = 1; metastatic tumor, N = 2; brain abscess, N = 1; encephalitis, N = 1; and radiation necrosis, N = 1). Among the analyzed markers, the expression of CD206, known as an M2 marker, was synchronously upregulated in pMG and iMG cells in six of the nine patients (67%) in the GLI group (**Figure 3A**). Upregulation of CD206 expression in pMG and iMG cells was particularly pronounced in individuals with GBM, as this phenomenon was observed in four of the five patients studied (80%). The characteristics of the patients from the GLI group are summarized in **Table 3**, revealing no clear predictive biomarker or background characteristics correlating with CD206 upregulation. In the RGM group, synchronous upregulation of CD206 expression was detected only in two (meningioma and brain abscess) of the six (33%) patients (**Figure 3B**). The brain tissue around the tumor of the patient with meningioma showed reactive astrocytes and edematous change probably because of the physical compression (**Supplementary Figure S2**). The surrounding tissue of the patient with brain abscess showed edematous change and infiltration of inflammatory cells. There was no increment in CD206 expression level in two patients with metastatic tumor and two patients who underwent removal of tumor-suspected lesions (radiation necrosis) and encephalitis suspected lesion. Altogether, CD206 upregulation in microglia shown in the surrounding tissue might be a hallmark of glioma, especially in GBM, and can be detected by isolating iMG cells from the peripheral blood of patients.

**Table 3:** Summary of the pathological characteristics and genetic mutations in patients from the glioma group.

Case No.	14	15	16	17	18	19	20	21	22
**Diagnosis**	DA	DA	AA	AG	GBM	GBM	GBM	GBM	GBM
**CD206 upregulation**	+	+	−	−	+	+	−	+	+
***ATRX***	loss	loss	loss	retained	retained	loss	retained	retained	retained
***Ki-67***	11.10%	2.40%	4.60%	19.50%	40.90%	36%	17.60%	56.40%	60.00%
***IDH1 or 2***	wild type	mutant	mutant	wild type	wild type	mutant	wild type	wild type	wild type
***BRAF***	wild type	wild type	wild type	wild type	wild type	wild type	wild type	wild type	wild type
***H3F3A***	wild type	wild type	wild type	mutant	wild type	wild type	wild type	wild type	wild type
***MGMT***	methylated	methylated	methylated	unmethylated	unmethylated	methylated	methylated	methylated	unmethylated
***TERT-p***	wild type	wild type	wild type	wild type	wild type	wild type	wild type	mutant	wild type
***EGFR***	wild type	gain	wild type	wild type	wild type	wild type	gain	gain	gain
***CDKN2A/B***	wild type	wild type	wild type	wild type	wild type	wild type	wild type	wild type	hemizygous deletion
***PTEN***	wild type	wild type	wild type	wild type	wild type	wild type	wild type	loss	loss
***p53***	wild type	heterozygous	wild type	loss	loss	heterozygous	loss	loss	loss
**chr10 LOH**	retained	retained	partial	retained	total	retained	retained	total	total
**CDK4**	wild type	wild type	loss	wild type	wild type	wild type	wild type	amplified	wild type
**PDGFRA**	wild type	wild type	wild type	wild type	wild type	wild type	wild type	amplified	wild type

DA, Diffuse astrocytoma (WHO grade II); AA, Anaplastic astrocytoma (WHO grade III); AG, Anaplastic ganglioglioma (WHO grade III); GBM, Glioblastoma (WHO grade IV).

### Expression CD206, Iba-1, and CD68 in Human Brain Tumor Samples

**Figure 4:**
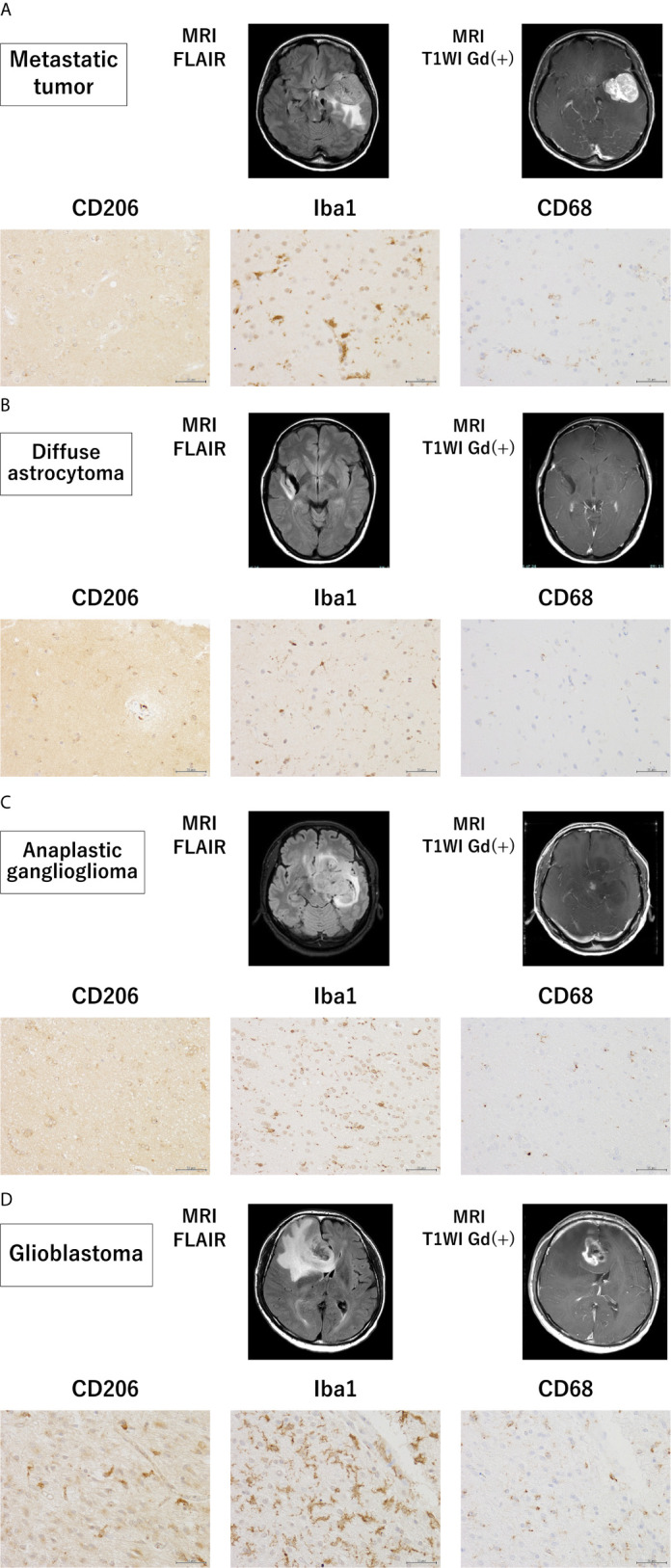
Magnetic resonance imaging findings and immunohistochemistry staining in samples from patients with grade II–IV glioma and metastatic tumor. Magnetic resonance imaging findings and representative immunohistochemical staining for CD206, Iba-1, and CD68 of the brain tissues surrounding a metastatic tumor **(A)** and glioma (WHO grade II–IV) **(B–D)** (scale bars = 50 µm).

To assess the invasion of microglia/macrophages in the brain tissue around the tumor, tissue samples of grade II–IV glioma and metastatic tumor were analyzed using immunohistochemistry (**Figures 4A–D**). Interestingly, the Iba1-positive GAM fraction was the most predominant in all tumors, followed by a slightly lower proportion of cells positive for CD68, a marker for the activated microglia. CD206 tended to be highly expressed in brain tissue surrounding GBM, and conversely, it was weakly expressed in all metastatic brain tumors and grade III gliomas. In grade II gliomas, CD206-positive GAM were present in high numbers. These results were consistent with the PCR results in all tumor cases.

## Discussion

Our study demonstrates that some immune properties of GAM can be monitored using iMG cells isolated from peripheral blood. In recent years, the development of drugs targeting tumor immunity has progressed rapidly. In particular, immune checkpoint inhibitors have already been confirmed to be effective in clinical trials for tumors such as melanoma (2); however, these drugs have not been proven effective for GBM. Mounting evidence has demonstrated that immune response is involved in the development and progression of GBM (14); therefore, a thorough investigation of the underlying molecular and immunologic mechanisms of GBM tumorigenesis is important for developing novel interventions. Our study demonstrated that the specific immune status of glioma might be monitored using peripheral iMG cells, which can be utilized as an effective research tool for the elucidation of immunologic mechanisms of tumorigenesis. Furthermore, GAM that play a role in supporting tumor invasion (35) may become a new target for immunotherapy of GBM in the future.

Our study revealed the specific upregulation of CD206 in iMG cells isolated from the peripheral blood of patients with glioma. These findings indicate that our technique can be used to develop a diagnostic marker for glioma. Although neuroradiological examinations, such as computed tomography and magnetic resonance imaging, are mainly performed as standard clinical examinations for the preoperative diagnosis of GBM, radiographic characteristics of GBM are similar to those of other neuronal diseases, including metastatic brain tumor, brain abscess, and primary CNS lymphoma. Therefore, the development of a tumor-specific marker for GBM would greatly facilitate the presurgical confirmation of diagnosis. There have been several recent attempts of molecular diagnosis of gliomas using cerebrospinal fluid (36, 37); however, those techniques have not achieved the detection of glioma-specific mutations in peripheral blood cells. Recently, several reports on serum microRNAs can distinguish patients with gliomas from healthy controls with high sensitivity and specificity (38, 39). Zhou et al. (40) reviewed 28 reports on the diagnosis of glioma using microRNAs and reported overall sensitivity of 85% and specificity of 90%. Although these studies suggested that microRNA levels are useful for distinguishing glioma and non-glioma cases, a complete consensus has not been reached to date. Our report provides a novel alternative approach for developing a non-invasive diagnostic tool for brain tumors based on a peripheral blood test.

We have previously reported that CD206 expression in iMG cells was downregulated in manic patients with bipolar disorder (34). We hypothesized that psychiatric states change microglial polarization and affect the immune environment, associated with changes in CD206 expression levels. The present study extends the significance of CD206 expression in iMG cells as a marker to glioma, another CNS disease.

Our study revealed the upregulation of CD206 expression using qRT-PCR of microglia extracted from brain tissues around GBM in four of the five studied cases. Few studies have reported that GAM are associated with the pathological grade and prognosis of gliomas. A positive correlation between the World Health Organization pathological grades and the expression of CD206-positive GAM has been reported in human glioma tumor tissues (21). GAM may be induced to M2 polarization by glioma-secreted factors, thereby supporting tumor invasion and growth (35). In low-grade gliomas, Lee et al. reported correlations between malignant transformation, *CX3CR1* V249I gene polymorphisms, and tumor immune microenvironment. Tumors from patients that were heterozygous or homozygous for *CX3CR1* V249I polymorphisms showed less infiltration of M2 macrophages and had a better prognosis than those in patients without these mutations (41).

We found that iMG cells derived from the peripheral blood showed similar CD206 profiles to pMG in the brain. Recently, some reports have shown that extracellular vesicles derived from GBM can change the phenotype of GAM to an M2-like anti-inflammatory phenotype (42, 43). Gabrusiewicz et al. showed that exosomes secreted from GBM stem cells mainly targeted peripheral blood monocytes to induce immune suppressive M2 phenotype by secreting cytokines such as monocyte chemotactic protein-3 and chemokine (C-X-C motif) ligand 1 (44). Thus, not only the interaction between GBM and GAM, but also between GBM and peripheral blood monocytes through humoral factors and/or extracellular vesicles may reflect the microenvironment of gliomas and can be detected by analyzing iMG cells derived from peripheral monocytes.

### Limitations

Studying human GAM has two major challenges. Control tissues are derived from post-mortem tissues or diseased non-tumor patients and, most notably, from epileptic patients because of the lack of naive control samples. However, the brain tissues obtained from patients with epilepsy are not completely normal. The second challenge is the current lack of reliable surface markers for distinguishing brain-resident microglia from infiltrating myeloid cells in the human brain. Experiments with functional assays and flow cytometry would be required to evaluate how closely iMG cells reflect the immunological activity of GAM. However, these experiments could not be performed in this study because the number of cells collected was limited. In several epilepsy patients, non-specific upregulation of microglial markers, including CD206, were observed in tissue samples obtained by the second look surgeries performed approximately 1 or 2 weeks after craniotomies for subdural electrode placements. Histopathological examination of such tissue samples revealed infiltration of chronic inflammatory cells in the subarachnoid space. Physical brain damage caused the infiltration of inflammatory cells, which was thought to have affected the upregulation of CD206 expression (45–47).

In this study, synchronous upregulation of CD206 expression levels was observed in most patients with glioma; however, patients with meningioma and brain abscess showed similar levels. Identifying differences in the properties of microglia and iMG cells between glioma, meningioma, and brain abscess is recommended for future studies. As recent attempts to identify markers distinguishing microglia and monocytes/macrophages have not reached any consensus (48, 49), we analyzed human data using a sorting method based on CD11b^+^ MACS: this approach assesses all myeloid cells, including monocytes, macrophages, dendritic cells, and neutrophils, besides microglia. Our findings were based on a limited number of patients with brain tumors, including GBM, and a limited number of epilepsy patients as control. Therefore, future studies with a larger number of patients will be necessary to confirm our results.

## Conclusion

In summary, our study revealed that peripheral iMG cells obtained by our previously developed technique can be used to gauge the properties of pMG from the tumor lesion microenvironment in the CNS. Therefore, iMG cells are novel, less-invasive tools for monitoring the disease-related immunological state of microglia. They can be used to investigate the roles of microglia in various brain diseases, including glioma. The upregulation of CD206 expression detected using iMG cells has the potential to be used as a glioma biomarker. Our study represents the first step towards understanding the contribution of GAM to the proliferation and invasion of GBM cells. Further studies are needed for investigating the role of microglia in GBM as a better understanding of GAM roles in GBM may provide a new therapeutic target for GBM treatment.

## Data Availability Statement

The original contributions presented in the study are included in the article/**Supplementary Material**, further inquiries can be directed to the corresponding author/s.

## Ethics Statement

The studies involving human participants were reviewed and approved by The ethics committee of the Graduate School of Medical Sciences, Kyushu University (application number: 26-406 and 29-624). Written informed consent to participate in this study was provided by the participants’ legal guardian/next of kin. Informed consent was obtained from the patients whose brain tissues had to be resected during the operative procedure for the treatment of brain diseases under a protocol approved by the Ethics Committee of the Graduate School of Medical Sciences at the Kyushu University (application number: 26-406 and 29-624). Freshly resected patient samples and blood samples were provided by the Department of Neurosurgery of the Kyushu University Hospital. Consent to collect blood was not obtained in three patients with epilepsy due to their young age and low body weight. Handling and analysis of these tissues were performed with the approval of the Ethical Committee and according to the principles of the Declaration of Helsinki.

## Author Contributions

ST, MO, NH, and TK designed the study. ST and MO conducted the experiments, analyzed the results, and wrote the manuscript. SI, NSa, NSh, YFuj, KT, and YFun performed the experiments. SS and HH analyzed the data. NH, NM, RH, YS, TI, MH, KI, MM, and TK critically revised the manuscript. All authors contributed to the article and approved the submitted version.

## Funding

This work was partially supported by Grants-in-Aid for Scientific Research from (1) The Japan Agency for Medical Research and Development (Syogaisya-Taisaku-Sogo-Kenkyu-Kaihatsu-Jigyo to TK (JP18dk0307075), Yugo-No to TK (JP19dm0107095), and MH (JP19ek0610015)), (2) KAKENHI - the Japan Society for the Promotion of Science (JP26713039, JP15K15431, JP16H03741, JP16H06403, JP18H04042 & JP19K21591 to TK, JP19K17065 to MO, JP20K09392 to NH, JP21H03044 to MM), and (3) SENSHIN Medical Research Foundation (to TK). The funders had no role in study design, data collection, and analysis, decision to publish, or manuscript preparation.

## Conflict of Interest

The authors declare that the research was conducted in the absence of any commercial or financial relationships that could be construed as a potential conflict of interest.

## Supplementary Material

Supplementary Figure 1 Negative control staining for CD 206, Iba-1, and CD68 in patients with grade II–IV glioma and metastatic tumor. The negative controls for CD 206, Iba-1, and CD68 of the brain tissues surrounding metastatic tumor **(A)** and glioma (WHO grade II–IV) **(B–D)** (scale bars = 50 µm).

Supplementary Figure 2 Hematoxylin and eosin staining of the brain tissue around the tumor of a patient with secretory meningioma. The brain tissue around the tumor of a patient with secretory meningioma showed reactive astrocytes and edematous change (scale bars = 50 µm).

## Abbreviations

CNS, Central nervous system; GAM, Glioma-associated microglia/macrophages; GBM, Glioblastoma; GLI group, Glioma group; GM-CSF, Granulocyte macrophage colony-stimulating factor; IL, Interleukin; iMG cells, Induced microglia-like cells; PBMCs, Peripheral blood mononuclear cells; pMG, Primary microglia; qRT-PCR, Quantitative reverse transcription-polymerase chain reaction; RGM group, radiographic glioma-like mass lesion group; TGF-β, Transforming growth factor-beta; TNF-α, Tumor necrosis factor-alpha.
