# Supplementary figures and images for: CD206 Expression in Induced Microglia-Like Cells From Peripheral Blood as a Surrogate Biomarker for the Specific Immune Microenvironment of Neurosurgical Diseases Including Glioma
Source: Front Immunol. 2021 Jun 29;12:670131. doi: 10.3389/fimmu.2021.670131 (PMC8276757; doi:10.3389/fimmu.2021.670131)

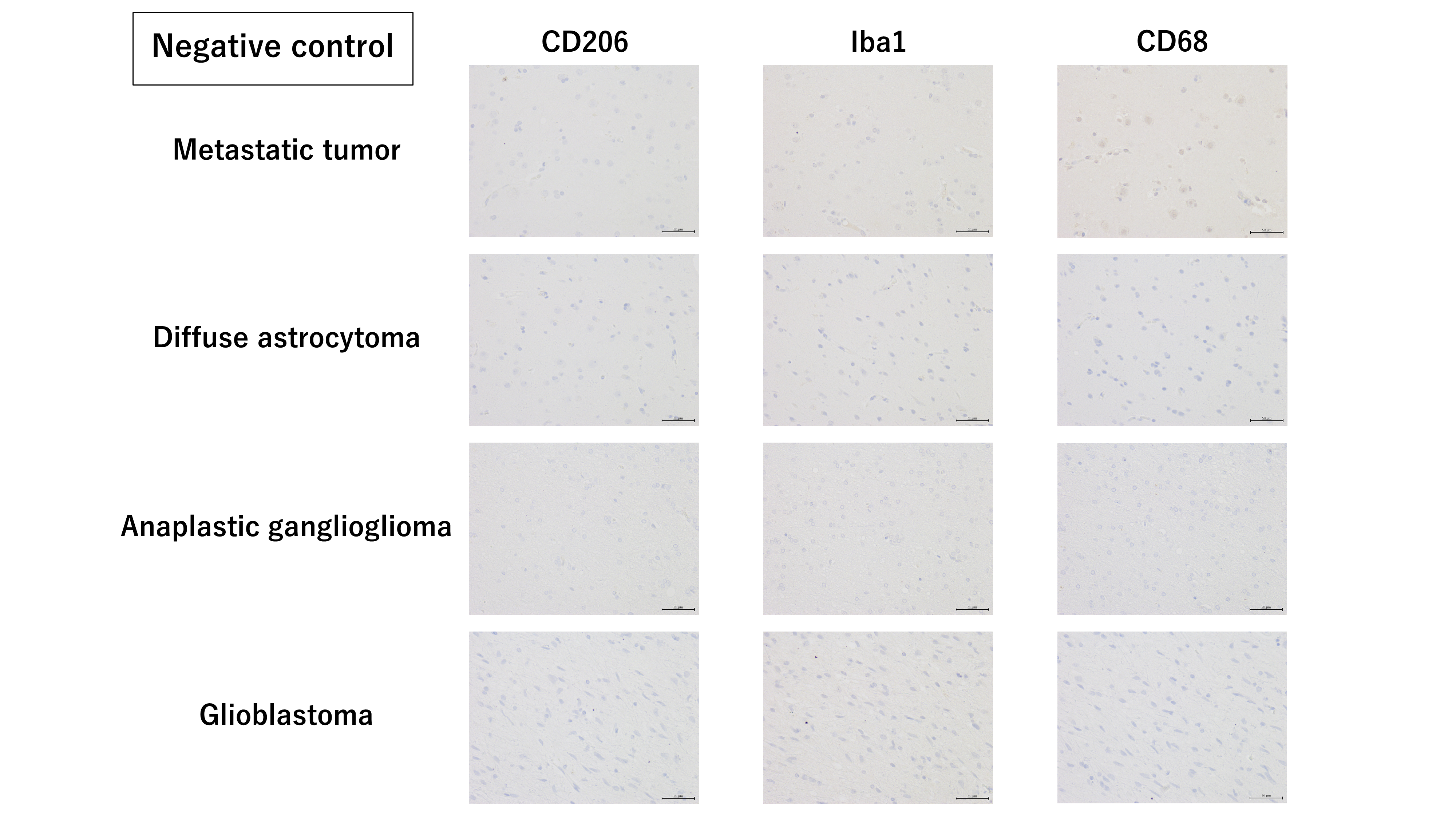

Supplement: Supplementary Figure 1 — Negative control staining for CD 206, Iba-1, and CD68 in patients with grade II–IV glioma and metastatic tumor. The negative controls for CD 206, Iba-1, and CD68 of the brain tissues surrounding metastatic tumor (A) and glioma (WHO grade II–IV) (B–D) (scale bars = 50 µm). [file Image_1.tif]

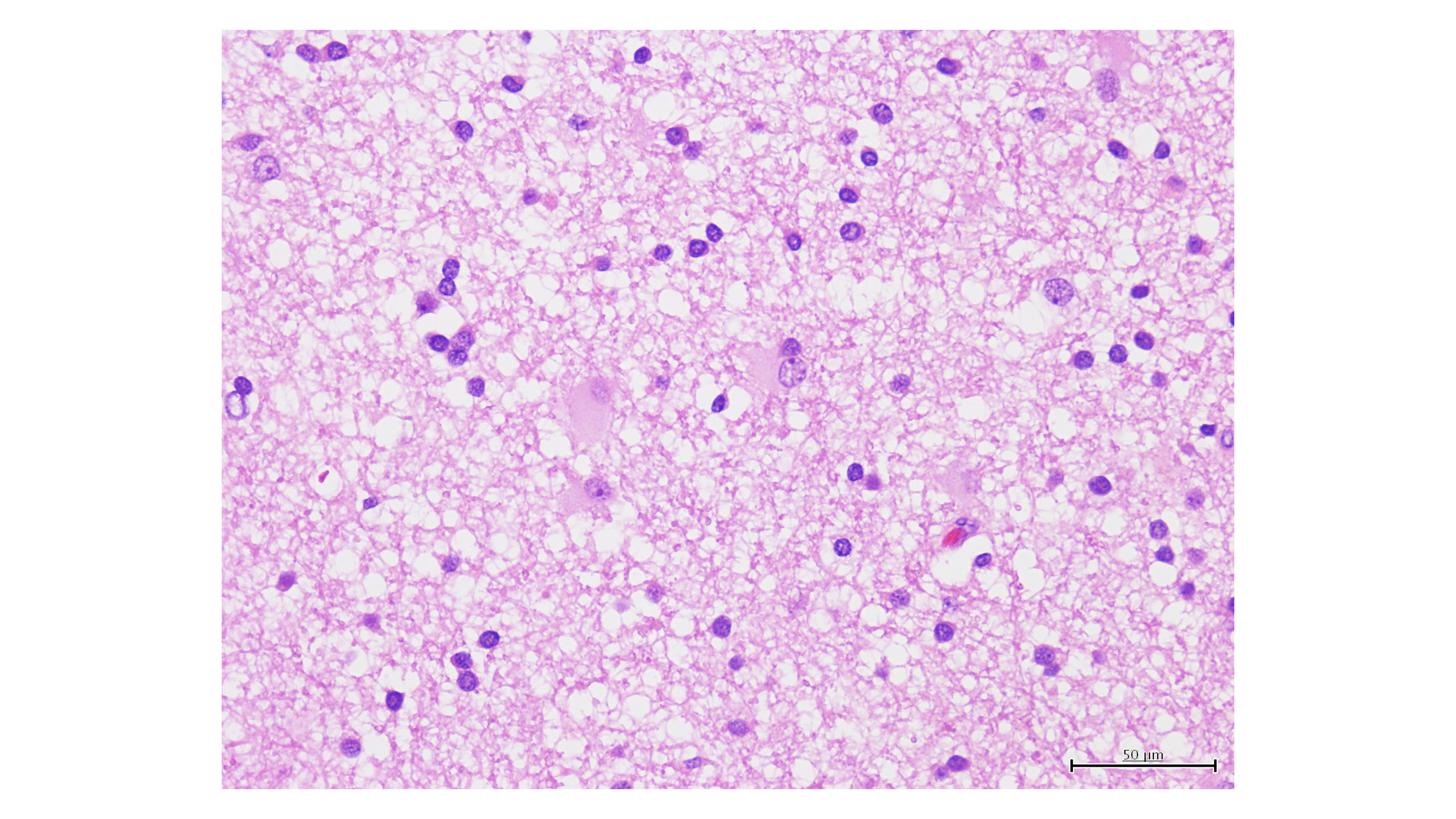

Supplement: Supplementary Figure 2 — Hematoxylin and eosin staining of the brain tissue around the tumor of a patient with secretory meningioma. The brain tissue around the tumor of a patient with secretory meningioma showed reactive astrocytes and edematous change (scale bars = 50 µm). [file Image_2.tif]
