# Supplementary material for: CD206 Expression in Induced Microglia-Like Cells From Peripheral Blood as a Surrogate Biomarker for the Specific Immune Microenvironment of Neurosurgical Diseases Including Glioma
Source: Front Immunol. 2021 Jun 29;12:670131. doi: 10.3389/fimmu.2021.670131 (PMC8276757; doi:10.3389/fimmu.2021.670131)
Supplement: Supplementary file 3 [file Table_1.docx]

|  | M1 marker | | | | | | | | | | | | M2 marker | | | | | | | | | | | |
| --- | --- | --- | --- | --- | --- | --- | --- | --- | --- | --- | --- | --- | --- | --- | --- | --- | --- | --- | --- | --- | --- | --- | --- | --- |
|  | TNFa | | CD45 | | IL-1b | | CD80 | | IL-23 | | HLA-DR | | BDNF | | CD206 | | CD209 | | IL-10 | | CCL18 | | CD23 | |
| case No. | pMG | iMG | pMG | iMG | pMG | iMG | pMG | iMG | pMG | iMG | pMG | iMG | pMG | iMG | pMG | iMG | pMG | iMG | pMG | iMG | pMG | iMG | pMG | iMG |
| 5 | 0.338 | 1.85 | 1.39 | 0.958 | 0.267 | 0.495 | 1.61 | 0.349 | 0.891 | 0.217 | 4.97 | 2.15 | 6.83 | 0.8 | 216 | 107 | 0.579 | 0.076 | 1.51 | 1.48 | 0.944 | 0.509 | 0.61 | 0.417 |
| 6 | 0.055 | 0.597 | 1.99 | 2.18 | 0.512 | 5.47 | 1.42 | 1.81 | 0.051 | 0.276 | 4.17 | 3.27 | 2.84 | 0.688 | 515 | 250 | 1.29 | 0.574 | 4.13 | 1.06 | 17.7 | 3.94 | 0.37 | 0.422 |
| 8 | 1.28 | 0.646 | 4.38 | 1.93 | 2.36 | 25.7 | 1.64 | 1.09 | 5.33 | 16.9 | 5.48 | 11.2 | 6.54 | 9.02 | 291 | 174 | 6.93 | 0.71 | 10.8 | 21.5 | 9.65 | 4.83 | 0.525 | 1.26 |
| 9 | 0.246 | 3.03 | 1.66 | 0.801 | 0.147 | 14.8 | 0.563 | 0.318 | 0.054 | 0.435 | 1.61 | 0.878 | 1.04 | 2.33 | 0.338 | 0.147 | 0.258 | 0.191 | 0.074 | 0.216 | 2.4 | 0.658 | 3.1 | 1.36 |
| 10 | 0.032 | 0.183 | 0.722 | 1.25 | 0.109 | 1.35 | 0.484 | 0.283 | 0.129 | 0.436 | 1.8 | 1.3 | 2.01 | 1.07 | 1.81 | 0.375 | 0.575 | 0.1 | 0.355 | 0.732 | 0.954 | 0.35 | 0.543 | 0.449 |
| 11 | 0.959 | 2.5 | 1.57 | 0.942 | 1.91 | 6.98 | 1.03 | 0.785 | 6.07 | 16.3 | 12.2 | 12.5 | 1.03 | 4.89 | 60.4 | 86.8 | 1.06 | 0.39 | 140 | 526 | 3.91 | 1.81 | 0.857 | 1 |
| 12 | 0.116 | 0.305 | 1.08 | 1.01 | 0.441 | 0.437 | 1.41 | 0.401 | 0.024 | 0.273 | 1.34 | 0.808 | 0.178 | 0.759 | 1.57 | 0.447 | 1.58 | 0.121 | 0.606 | 0.329 | 2.29 | 0.44 | 1.35 | 0.443 |
| 13 | 1.22 | 0.248 | 1.88 | 0.65 | 2.26 | 8.19 | 0.358 | 0.952 | 6.63 | 8.72 | 13 | 4.33 | 0.159 | 2.47 | 0.444 | 0.414 | 0.628 | 0.482 | 11.8 | 19.5 | 0.347 | 0.814 | 0.689 | 0.725 |
| 14 | 1.37 | 0.215 | 1.43 | 1.48 | 1.83 | 0.218 | 2.35 | 0.204 | 4.19 | 0.605 | 0.692 | 2.06 | 4.45 | 0.54 | 178 | 493 | 1.99 | 0.049 | 0.794 | 2.22 | 0.844 | 0.294 | 0.408 | 0.436 |
| 15 | 1.79 | 1.25 | 1.94 | 0.735 | 2.03 | 28.3 | 12.2 | 7.65 | 22.1 | 21.9 | 3.15 | 1.41 | 2.3 | 1.52 | 121 | 188 | 5.05 | 1.17 | 12.1 | 2.01 | 22.8 | 2.87 | 0.534 | 0.474 |
| 16 | 0.081 | 0.392 | 1.3 | 0.846 | 0.241 | 6.78 | 0.926 | 3.27 | 0.069 | 0.087 | 0.396 | 2 | 0.724 | 1.61 | 1.5 | 0.148 | 1.1 | 0.437 | 0.28 | 0.537 | 0.039 | 0.691 | 0.659 | 1.38 |
| 17 | 0.628 | 0.075 | 1.67 | 1.61 | 1.88 | 0.096 | 0.386 | 0.166 | 0.15 | 0.31 | 2.44 | 1.69 | 0.251 | 1.63 | 0.391 | 1.31 | 0.374 | 0.032 | 0.357 | 3.02 | 1.2 | 0.231 | 0.381 | 0.411 |
| 18 | 0.688 | 0.489 | 2.44 | 0.847 | 0.298 | 4.88 | 0.295 | 0.39 | 0.06 | 0.299 | 1.03 | 2.74 | 1.43 | 0.256 | 73.9 | 346 | 0.708 | 0.14 | 35.1 | 0.352 | 15.5 | 1.44 | 1.37 | 1.16 |
| 19 | 0.441 | 0.368 | 0.995 | 1.5 | 0.26 | 0.684 | 0.663 | 0.132 | 1.29 | 1.02 | 1.29 | 0.03 | 4.57 | 1.71 | 33.3 | 146 | 0.799 | 0.157 | 0.366 | 1.18 | 2.15 | 0.839 | 0.653 | 0.414 |
| 20 | 0.021 | 0.464 | 1.68 | 3.46 | 0.14 | 2.8 | 0.512 | 1.99 | 0.031 | 0.582 | 2.61 | 3.5 | 1.73 | 7.94 | 1.72 | 2.93 | 0.053 | 0.08 | 0.556 | 1.89 | 2.2 | 1.62 | 0.362 | 0.398 |
| 21 | 0.094 | 0.304 | 2.03 | 1.71 | 0.26 | 1.35 | 0.839 | 0.264 | 0.837 | 0.073 | 4.63 | 2.99 | 2.21 | 1.06 | 73.1 | 237 | 0.382 | 0.556 | 2.38 | 1.06 | 4.1 | 4.28 | 0.553 | 0.434 |
| 22 | 1.08 | 0.575 | 2.58 | 0.839 | 1.79 | 2.74 | 2.73 | 0.665 | 4.17 | 3.35 | 13.3 | 5.53 | 8.51 | 1.34 | 300 | 397 | 4.7 | 1.22 | 50.2 | 164 | 17.4 | 0.44 | 0.883 | 0.681 |

Supplementary Table S1. Results of qRT-PCR for representative M1 and M2 microglial markers in pMG and iMG cells

mRNA levels of all M1 and M2 markers (12 types in total) were measured using qRT-PCR in all 19 cases in which mRNA could be extracted from both induced microglia-like (iMG) cells and primary microglia (pMG). Each value was compared with the control (epilepsy cases without inflammatory infiltrates due to subdural electrode placements, N = 2) to investigate the correlation between iMG cells and pMG.
